# Transcriptomic portrait of human Mesenchymal Stromal/Stem cells isolated from bone marrow and placenta

**DOI:** 10.1186/1471-2164-15-910

**Published:** 2014-10-19

**Authors:** Beatriz Roson-Burgo, Fermin Sanchez-Guijo, Consuelo Del Cañizo, Javier De Las Rivas

**Affiliations:** Bioinformatics and Functional Genomics Group, Cancer Research Center (IBMCC, CSIC/USAL/IBSAL), Consejo Superior de Investigaciones Cientificas (CSIC), Salamanca, Spain; Hematology Department, University Hospital of Salamanca (HUS, IBSAL/USAL), Salamanca, Spain

**Keywords:** Stromal cells, Mesenchymal stem cells, Placenta, Bone marrow, Human gene expression, RNA-Seq, Transcription factors

## Abstract

**Background:**

Human Mesenchymal Stromal/Stem Cells (MSCs) are adult multipotent cells that behave in a highly plastic manner, inhabiting the stroma of several tissues. The potential utility of MSCs is nowadays strongly investigated in the field of regenerative medicine and cell therapy, although many questions about their molecular identity remain uncertain.

**Results:**

MSC primary cultures from human bone marrow (BM) and placenta (PL) were derived and verified by their immunophenotype standard pattern and trilineage differentiation potential. Then, a broad characterization of the transcriptome of these MSCs was performed using RNA deep sequencing (RNA-Seq). Quantitative analysis of these data rendered an extensive expression footprint that includes 5,271 protein-coding genes. Flow cytometry assays of canonical MSC CD-markers were congruent with their expression levels detected by the RNA-Seq. Expression of other recently proposed MSC markers (CD146, Nestin and CD271) was tested in the placenta samples, finding only CD146 and Nestin. Functional analysis revealed enrichment in stem cell related genes and mesenchymal regulatory transcription factors (TFs). Analysis of TF binding sites (TFBSs) identified 11 meta-regulators, including factors KLF4 and MYC among them. Epigenetically, hypomethylated promoter patterns supported the active expression of the MSC TFs found. An interaction network of these TFs was built to show up their links and relations. Assessment of dissimilarities between cell origins (BM versus PL) disclosed two hundred differentially expressed genes enrolled in microenvironment processes related to the cellular niche, as regulation of bone formation and blood vessel morphogenesis for the case of BM-MSCs. By contrast genes overexpressed in PL-MSCs showed functional enrichment on mitosis, negative regulation of cell-death and embryonic morphogenesis that supported the higher growth rates observed in the cultures of these fetal cells and their closer links with development processes.

**Conclusions:**

The results present a transcriptomic portrait of the human MSCs isolated from bone marrow and placenta. The data are released as a cell-specific resource, providing a comprehensive expression footprint of the MSCs useful to better understand their cellular and molecular biology and for further investigations on the isolation and biomedical use of these multipotent cells.

**Electronic supplementary material:**

The online version of this article (doi:10.1186/1471-2164-15-910) contains supplementary material, which is available to authorized users.

## Background

### Human adult Mesenchymal Stromal/Stem Cells (MSCs)

Adult stem cells retain the capacity for self-renewal and potential to differentiate into multiple specialized cell types. MSCs, discovered about 50 years ago [1], have been investigated in an effort to demonstrate their stem cell capabilities. In the late 1990s, colony-forming and plastic-adherent cells with fibroblast-like morphology isolated from human bone marrow (BM) were expanded *in vitro* and differentiated through mesodermal lineages such as osteoblasts, chondroblasts and adipoblasts [2, 3]. Over the last decade, other human organs have also emerged as hosts for MSC-like populations: muscles, tendons, skin, lungs, adipose tissue, umbilical cord, and placenta (PL) [4, 5]. Ease of access to some of these tissues (e.g. extra-embryonic annexes), together with their potential to regenerate damaged tissues and modulate the immune response, has triggered many clinical trials to assess the use of MSC in cell and tissue regenerative medicine [6, 7].

Molecular characterization of MSC phenotype has been elusive since a broad variation in the expression of different cluster of differentiation (CD) marker molecules has been shown [8, 9]. To date none of these markers has been found to be exclusive to MSCs, hampering the isolation of homogeneous primary cell populations. Moreover, several MSC subpopulations isolated from identical or alternative tissue sources have exhibited non-uniform cell differentiation potential [10]. To address this shortcoming, the *International Society for Cell Therapy* (ISCT, http://www.celltherapysociety.org/) has proposed that the MSCs can be identified by the expression of CD105, CD73 and CD90, and should be negative for the haematopoietic lineage markers CD45, CD34, CD14 (CD11b), CD19 and HLA-DR [11]. This combination of positive and negative CDs is widely accepted as a method for identifying human MSCs. However, a large genome-wide molecular characterization of the cellular phenotype is needed to properly determine MSC identity.

Here we analyze and compare MSC populations isolated from human bone marrow (BM-MSCs) and from placental tissue (PL-MSCs). Six primary cultures isolated from independent donors were subjected to comprehensive gene expression analysis using RNA deep sequencing (RNA-Seq). In this way, we make available here a detailed transcriptomic portrait of the human MSCs. An extensive analysis of the expression profiles obtained enabled us to map stem cell related genes and master transcription factors. As far as we know, there is not such large-scale data available up to date, providing a valuable resource to achieve a better characterization of MSCs and to help further future investigations.

## Methods

### Isolation of BM- and PL-MSCs

All the procedures performed in the current study were in accordance with the Declaration of Helsinki and all human samples were collected after signed informed consent was obtained as formally approved on June 16th of 2008 by the Ethics Committee of the Health Area of Salamanca (that provides appropriate ethical framework to the research performed at the University Hospital of Salamanca HUS and the Cancer Research Center IBMCC).

Human BM- and PL-MSCs from six healthy independent donors were expanded *in vitro*. The placental samples correspond to three healthy newborn girls. These samples were taken postpartum, immediately after delivering. The bone marrow samples correspond to three adult healthy donors of ages 41, 42 and 61 (two males and one female).

Placental chorionic sections (dissections from the fetal part of the placenta, i.e. the *Chorion frondosum*, 80 to 100 g weight) were collected in aseptic conditions just after parturition [12]. Each sample was washed thoroughly in normal saline solution, dissected into pea-sized fragments and enzymatically digested in 250 ml DMEM-LG medium (Gibco, Invitrogen), with 100 U/ml Collagenase type I (Gibco, Invitrogen) and 5 μg/ml DNase I (sterile, Roche). The mixture was incubated in a shaker for 2 h, at 37°C [13, 14]. Cell suspensions were filtered through 70 μm strainers (Becton Dickinson), centrifuged (300 × g, 10 min, 20°C), resuspended in Hanks Solution (Gibco, Invitrogen), and processed for mononuclear fraction separation (MNCs). Bone marrow samples of 10 to 20 ml from iliac crest aspirates were taken under local anesthesia under institutional standards [15]. MNCs were separated by density gradient centrifugation using Ficoll-Paque® (GE Healthcare Bio-Sciences), then seeded on a plastic surface (10^6^ MNCs/cm^2^) with DMEM-LG supplemented with 10% FCS (BioWhittaker, Lonza) and 1% penicillin/streptomycin (Gibco, Invitrogen) [16]. Cells were allowed to adhere for 3–5 days in a 37°C, 5% CO_2_ atmosphere. Thereafter, medium was completely changed twice a week. When confluence was reached, adherent cells were trypsinized (Trypsin-EDTA, Gibco, Invitrogen) and replated for culture expansion (seeding at 3,000-5,000 cells per cm^2^) [17]. Cell counts were performed with each passage. Population doubling times from first to sixth pass were assessed. Wilcoxon test searched for significant differences.

### Differentiation assays

MSCs were plated and grown with each specific differentiation media (from Miltenyi Biotec). For osteogenic and adipogenic capacities, MSCs were adhered to 9.6 cm^2^ slide flasks (Nunc, Roskilde). Alkaline phosphatase activity was evaluated by NBT/BCIP colorimetric reactions (nitroblue tetrazolium chloride/5-bromo-4-chloro-3-indolyl-phophate) (Roche). Adipogenesis was observed by Oil-Red-O staining of lipid vacuoles (Certistain Merck KGaA). Pelleted cells placed in conical tubes were also conditioned towards chondrogenic differentiation. The resulting cells were embedded in paraffin, cut into 5 mm sections and Hematoxylin-Eosin stained for evaluation of cartilage matrix formation [18].

### Immunophenotype characterization

MSC phenotypes, as defined in the ISCT minimal criteria [11], were tested by flow cytometry. MSCs (~10^6^ cells) were harvested, resuspended in PBS, and incubated with conjugated antibodies using the following panel: CD90-FITC, CD14-PE, CD45-PerCP/CD34-FITP, CD73-PE, HLA-DR-PerCP/CD44-FITC, 166-PE, CD19-PerCP, CD105-APC/CD11b-FITC, CD33-PE, 7AAD-PerCP (FITC: fluorescein isothiocyanate, PE: phycoerythrin, PerCP: peridinin chlorophyll protein, APC: allophycocyanin; Becton Dickinson Biosciences). 100,000 cell events per culture were acquired in a FACSCalibur flow cytometer (BD Biosciences) connected to the *CellQuest* program (BD Biosciences). Fluorescence-based expression of CD markers per event was analysed using *Infinicyt* software (Cytognos).

### RNA-Seq data production and processing

Two aliquots of 1–2 million MSCs per culture, from 3 BM and 3 PL samples in third passage, were lysed and frozen in TRIzol reagent (Invitrogen). Total RNA was isolated with chloroform and precipitated by centrifugation in isopropanol. DNA depletion was also performed. Poly-A mRNA selection and synthesis of a cDNA library were carried out following the Illumina TruSeq protocol. Single-end 105 bp length sequencing was performed on an Illumina GAIIx machine. Obtained reads were mapped against the HS19/GRCh37 reference genome using GSNAP (v. 2011-03-28) gapped-alignment algorithm [19]: up to 5 mismatches permitted; splicing junctions annotated from Ensembl 63. Quality controls of the sequencing process produced by the FastQC program (http://www.bioinformatics.babraham.ac.uk) were also evaluated. To visualize the aligned reads, bigWig format files were uploaded into the UCSC genome browser [20]. Specific genome locations of marker genes were zoomed in (data presented as the natural logarithm of the number of mapping reads, ranging from 0 to 10). Assembly and abundance of transcripts from uniquely mapping reads were conducted using two software tools: **(1)***Cufflinks* 1.0.3 [21], where FPKM values (fragments per kilobase of transcript per million mapped reads) were calculated with only reference-based assembled transcripts; and **(2)***htseq-counts* 0.4.7p2, for read count quantification, intersection-nonempty mode assembly was used as described in (http://www-huber.embl.de/users/anders/HTSeq/doc/count.html). The raw sequencing data files in fastq format are provided via *Galaxy* (http://galaxyproject.org/) at link: https://usegalaxy.org/u/cic19/h/mesenchymal-stem-cells-rnaseq.

*Cufflinks* summarized FPKM values per tissue were extracted with the *Cuffcompare* tool. Tissue *summarized log2(FPKM)* were calculated and plotted facing BM-MSC against PL-MSC. Similarly, the *means of log2(FPKM)* per tissue were also computed. Using the density distribution of *log2(FPKM)* we set up a cut-off value of 1 to separate two major components in the data distribution. For differential expression tests, *Cuffdiff* from Cufflinks software and *DEseq* package from R/Bioconductor [22] were applied over FPKM values and read-counts respectively. Significant genes were selected using multiple-test adjusted p-values [23]. R statistical computing environment, version 2.13.0 (http://www.r-project.org), was used for data management and for most calculations.

### Functional analysis

Several reference gene-sets were recruited and mapped on the MSC expression scatter: **(i)** a set of 158 curated housekeeping genes appearing in several datasets [24, 25], **(ii)** a list of 299 stem cell related genes taken from Loring lab [26]; **(iii)** a list of 740 known human transcription factors derived from TcoF-DB (cbrc.kaust.edu.sa/tcof) [27] and from the census of human TFs done by Vaquerizas *et al.*[28]. Ensembl identifiers were used for cross-reference all lists. Significant enrichment was tested using hypergeometric tests and tools from the *HTSanalyze* R-package. DAVID bioinformatics tool was also used for functional annotation enrichment and clustering [29].

### Transcription Factor Binding Sites (TFBS) analysis on expressed genes

Promoter and regulatory regions of the expressed genes were analysed exploring their DNA sequences from −2000 and −1 bp upstream the Transcription Start Site (TSS), from −5000 to −800 bp and also from −5000 to 200 bp inside of the genes. TFBSs that mapped into these regions were tested for over-representation. Databases mined for PSSMs (position-specific scoring matrices) were: JASPAR Core for Vertebrata [30], TRANSFAC (version 2009.4) [31] and *UniProbe*[32]. The analyses were done using three tools: **(1)***matrix-scan* from *RSAT*[33]; **(2)***TransFind*[34]; and **(3)***oPOSSUM*[35]. The dataset of 5,271 genes, and the 135 TFs included, were analysed to find *Cis*-regulatory modules. Results were summarized in a contingency table, where TFBSs were positively counted when found significantly enriched for each method. Not-assigned (NA) was indicated when corresponding PSSMs were not available for a given method. Specific parameters related to each run of the TF set analysis are detailed in `nal file 1: Table S4. Analysis with *RSAT* of random sets including 135 genes (called RRS) were added as negative controls. If any TFBS was found significant with the RRS, a −1 penalty was given. Experimentally-proven protein-protein interactions (PPIs) between TFs were obtained using APID (http://bioinfow.dep.usal.es/apid/) [36] and APID2NET [37]. Networks were built using Cytoscape 2.6.3 (http://www.cytoscape.org). Edge thickness and number indicates experimental evidence supporting each interaction. Colour fractions inside each node show the protein domains obtained from InterPro (http://www.ebi.ac.uk/interpro/).

### DNA methylation data

An external dataset of BM-MSCs from the GEO (GSE34688 [38]) including DNA methylation levels –measured with Illumina HumanMethylation450 BeadChip– was analysed. The normalized methylated and un-methylated signals of CpG sites were stored in a MethylSet object using *minfi* R-package [39]. Beta values of CpGs were calculated with the *getBeta* function, based on Illumina’s standard (Beta = M/(M + U + 100)). CpGs with detection p-values greater than 0.01 were filtered out. The median of Beta values across samples was used in graphic reports. Three random sets of 135 TFs not expressed in MSCs were selected as negative control sets. CpG island regions were defined as in [40], using the UCSC identifiers and RefSeq genes from the platform annotation file. Wilcoxon statistic was used to test significant differences between Beta value distributions.

## Results

### Isolation and culture of mesenchymal stromal cells

**Figure 1:**
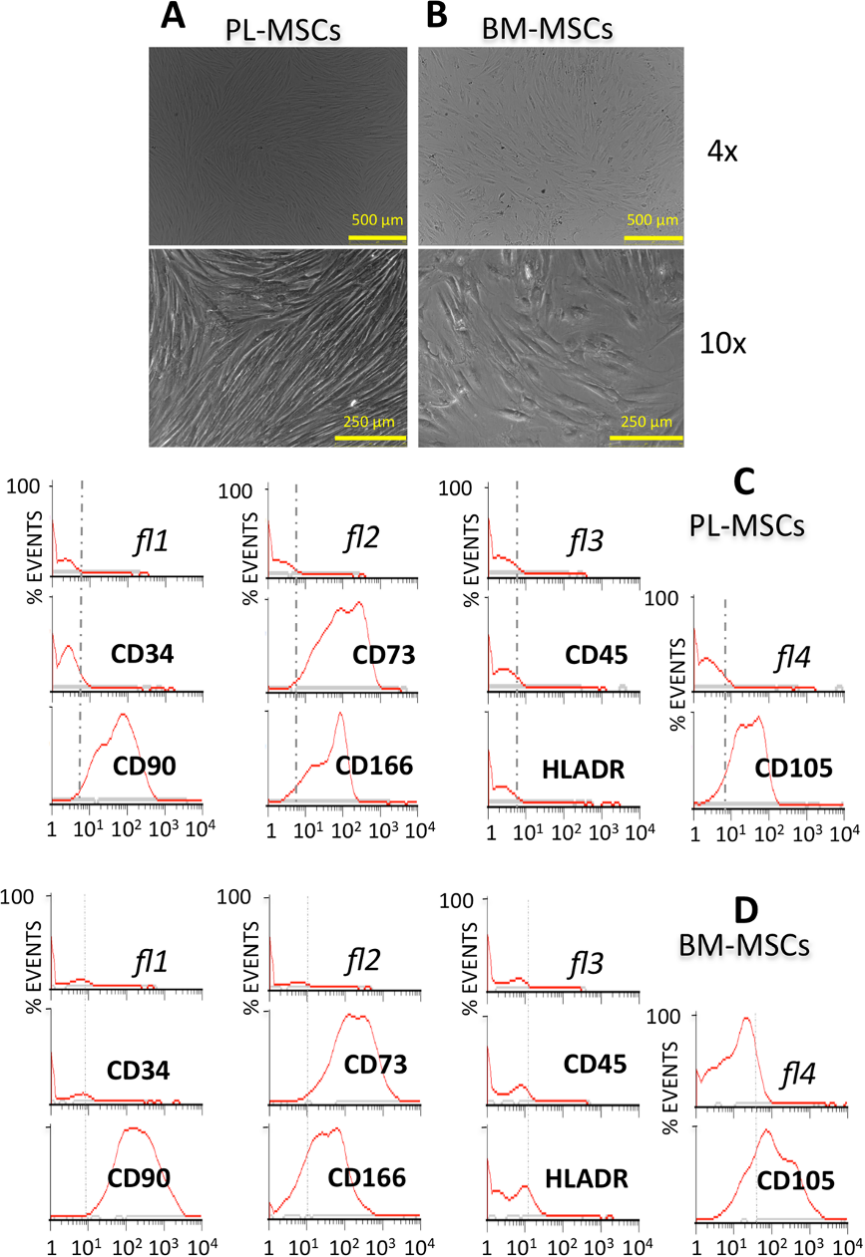
**Characterization of MSCs primary cultures: microscope and flow cytometry.** Microscope photographs of human MSCs in culture isolated from PL **(A)** and BM **(B)**: phase contrast micrographs of passage three cultures seen at two amplifications. Flow cytometry histograms of standard immunophenotype markers (CD34, CD73, CD45, CD90, CD166, HLADR, CD105) tested in isolated PL **(C)** and BM **(D)** MSCs.

Primary cultures of human MSCs were derived from two sources: chorionic placenta (PL) and bone marrow (BM). In both cases, adherent cells expanded *in vitro* until passage 3 displayed morphologic and molecular characteristics that define the MSCs (Figure 1A and 1B). Their ability to differentiate into osteoblasts, adipocytes and chondrocytes were proven in at least three independent samples from each origin (Additional file 2: Figure S1). Flow cytometry data showed positive expression of CD90, CD73 and CD105 in all third-passage populations (94.36 ± 6% of events acquired) (Figure 1C and 1D). Haematopoietic lineage markers CD34, CD45, CD19 and HLA-DR turned out to be negative throughout all third-passage populations, verifying the accepted criteria for the MSCs defined by the ISCT [11]. CD166 and CD44 markers showed low to medium expression, higher in PL-MSCs than in BM-MSCs. Finally, confluent cells exhibited slight differences: PL-MSCs seemed narrower than BM-MSCs resembling a more acute spindle, reaching a higher degree of confluence and optimizing occupation of the available room (Figure 1A). The division rate was also higher for PL-MSCs (population doubling time: PL = 3.1 days; BM = 8.4 days; difference *p-value* = 0.000359).

### RNA levels of CD markers

**Figure 2:**
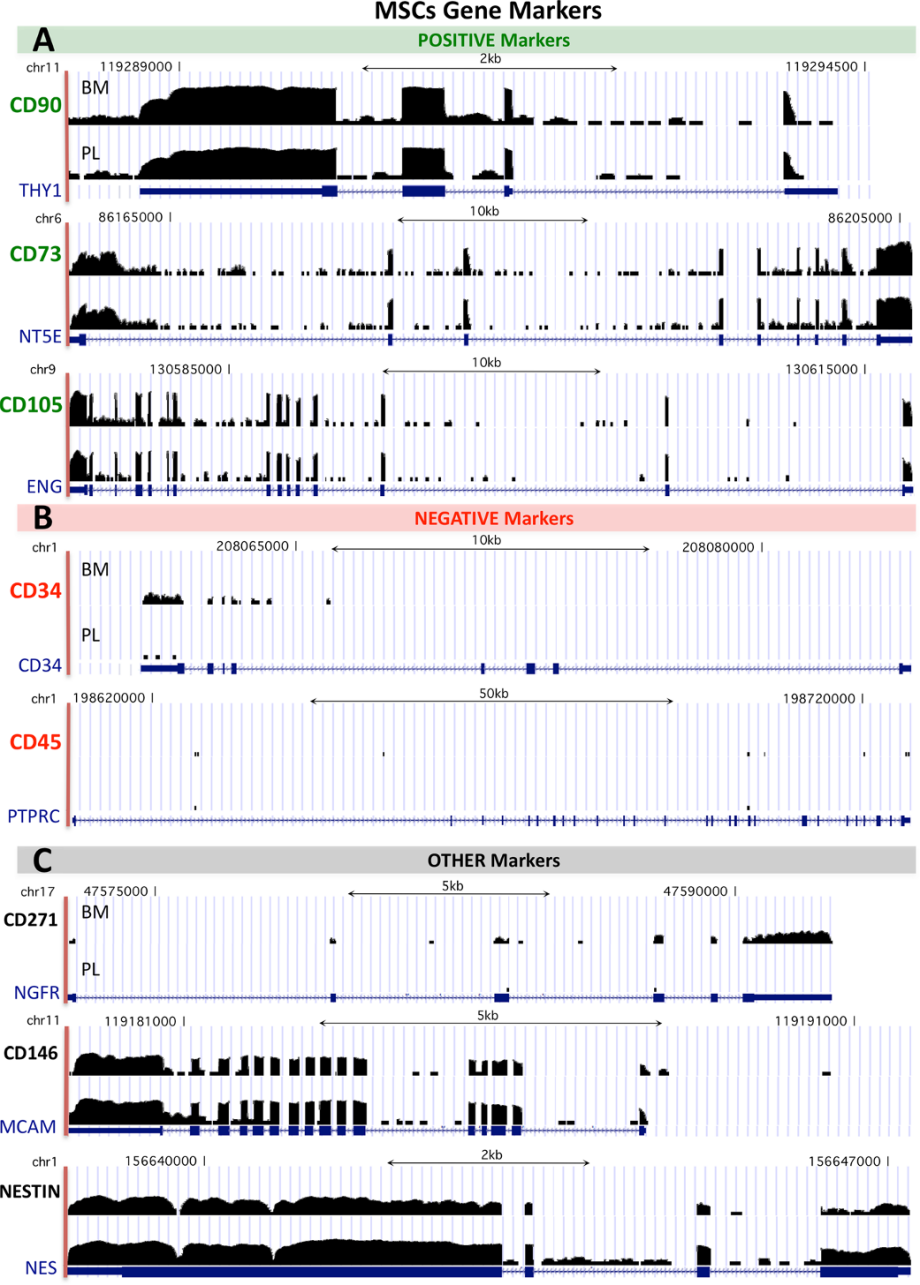
**MSCs RNA-Seq reads mapping on the loci of 8 specific genes.** A superimposed view piling-up the uniquely aligned RNA-Seq reads over the DNA regions of 8 specific gene loci, showing the exonic and intronic transcription outcomes. The expressed regions appear as black densities representing the raw number of reads on each specific region of the locus (transformed to log scale). The locus corresponding to each gene is indicated by the chromosome and the nucleotide number position, including scales bars in kb. For each gene locus the signal of BM-MSCs and PL-MSCs are placed one on top of the other, and the corresponding structure of the locus from RefSeqGene (NCBI) is painted below in blue. The genes are divided in **(A)** positive markers, **(B)** negative markers and **(C)** other markers.

Purified mRNA isolated from 3 BM- and 3 PL-MSC primary cultures from independent individuals was sequenced using Illumina-Solexa RNA-sequencing platform. Each average sample yielded a plethora of 42.14 million reads. In order to calculate expression signals, misaligned reads were avoided. In this way, a fraction of the reads uniquely aligned to the genome (90.17%) was retained for subsequent analysis (see details at Additional file 3: Figure S2). The number of reads obtained by a sequencing process is referred as the sequencing coverage, and the higher it is, the better it qualifies for a precise measure of expression levels [41]. The coverage along some MSC marker genes is shown in Figure 2, that presents on each gene locus the raw number of reads (log-transformed) provided by the RNA-Seq data (i.e. the reads on each specific region of the locus, exons or introns). This is a way to provide a view *in situ* of the original RNA-Seq signal, since in these graphical representations the black densities show the expression on each section of each gene locus. All exonic regions of markers CD90, CD73 and CD105 presented high read peaks in contrast to their intronic sequences. Some background signal might be detected in intronic regions as a result of detecting some immature mRNA. Mapping of reads over coding regions of negative markers (CD34, CD45) is practically nonexistent (Figure 2B). Thus, the phenotype of MSC protein markers previously assayed by flow cytometry presented coherent transcript expression levels.

Together with the well-known MSC markers, experimental assays using *in situ* hybridization have revealed other molecules as potential determinants of different human MSC subpopulations, such as: Nestin [42, 43], CD146 [44, 45] and CD271 [46, 47]. These markers have been originally assigned within the hematopoietic niche in BM-MSCs, and they may differ considerably between tissue types. Our results show homogeneous high transcription levels of CD146 (that is MCAM, melanoma cell adhesion molecule). Nestin (NES, an intermediate filament protein found in neuroepithelial stem cells) is also expressed in both PL- and BM-MSCs. It is worth to notice that in both cases (CD146 and Nestin) the expression in placenta is slightly higher than in bone marrow. Finally, CD271 (NGFR) does not show positive levels in any of the PL-MSC samples and it is quite low in the BM samples (Figure 2C).

### MSC gene expression footprint

**Figure 3:**
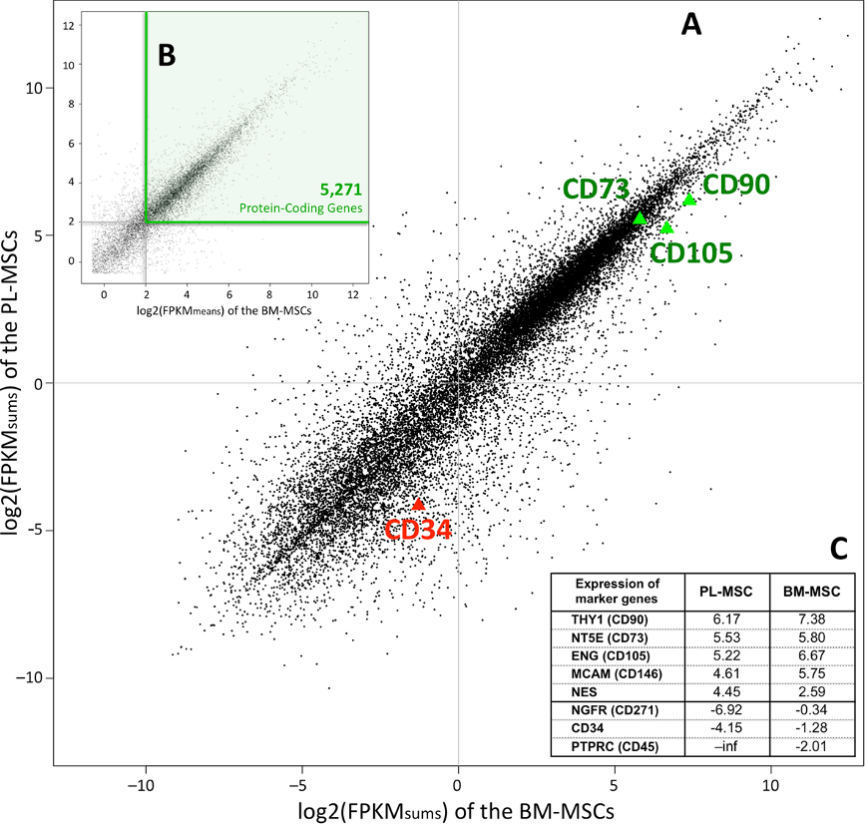
**Global expression: RNA-Seq expression data of human MSCs. (A)** Scatter plot presenting the values of *log2 (FPKM*_*sum*_ *)* for each gene in the BM-MSC samples (X-axis) versus the PL-MSC samples (Y-axis). Insert **(B)**: Scatter plot including the *log2 (FPKM*_*means*_ *)* of the protein-coding genes in the BM- and PL-MSC samples (showing with a green shade the region that includes 95% of the expressed data distribution). Insert **(C)**: Table indicating the *log2 (FPKM*_*sum*_ *)* values correspoding to 8 marker genes in the BM- and PL-MSC samples.

As indicated in Methods, the RNA sequenced reads mapped to the human genome were assembled per gene and condensed into FPKM expression values using *cufflinks* software (cufflinks.cbcb.umd.edu). These values provided a sample-centred absolute measure of the expression level of each gene in the studied cell population [21]. Reads from the all BM sample replicates were accounted together and compared against the equivalent from the PL samples. Summarized FPKM expression values of both types of MSCs can be represented on a scatter plot (*log2* scale of FPKM_sums_) (Figure 3A). This scatter representation of the FPKM signals of both subtypes of MSCs shows a strong linear correlation between them (Figure 3A), indicating a clear expression similarity, with a higher overlapping for values above 0. Most of the genes run along the diagonal and can be considered common genes, expressed similarly in both MSC subtypes. The CD cytometric positive markers (CD73, CD90 and CD105) were found centered at the top-right square of this expression scatter plot (quite close to the diagonal, with values >5). By contrast, the negative CD markers were detected below the 0 line (CD34) or not detected within the scale range (CD45). Therefore, the main density peak placed in the top-right square of the scatter plot (Figure 3A) includes the genes most likely expressed in MSCs. Narrowing the study to find a common consistent expression pattern of both MSC classes, the mean FPKM values of protein-coding expressed genes (across all six RNA-Seq samples) were calculated for the region above 0 (i.e. for *log2*(FPKM_means_) > 0, that is FPKM > 1) (Figure 3B). Analysis of these means data distribution allowed us to set-up a cut-off corresponding to *log2*(FPKM_means_) ≥ 2 that included 95% of the data distribution and excluded the lowest 5% values. As indicated above, this distribution was produced considering only *log2*(FPKM_means_) > 0. To have an estimation of the amount of mRNA that these thresholds represent, value *log2*(FPKM_means_) = 2 corresponds to 4 FPKM mean signals per gene loci. Mortazavi *et al.*[48] reported that 3–4 RPKM corresponded to about one transcript count per cell when quantifying transcriptomes by RNA-Seq. Therefore, the selected cut-off of *log2*(FPKM_means_) ≥ 2 is adequate to determine genes that are truly expressed having at least one mRNA copy per cell. The region above this cut-off included a total of 8,534 genes (gene list with expression values provided in Additional file 4: Table S1; and in Additional file 5 in txt format), which corresponded to 5,271 protein-coding genes (Additional file 6: Table S2; and Additional file 7 in txt format). In this way, we obtained the set of genes that constitute the transcriptomic footprint of the human MSCs from two separated tissue origins. We focused on the protein-coding genes in this work because they provide the most accessible biological functions, although the ncRNAs included in the footprint should be also considered as essential part of the MSCs trascriptome depicted.

### Comparison of global expression profiles of MSCs with other related cell-types

The transcriptomic profiling presented was derived from the analyses of deep coverage RNA-Seq datasets from three independent biological replicates of two human MSCs from different origin (bone marrow and placenta). Each independent mRNA sequencing provided more than 36 millions of uniquely-mapped reads per sample (Additional file 3: Figure S2) and the consistency of the replicates in the expression signal quantification per locus was >99% in the reported MSC gene footprint. In a separate transcriptomic study other related multipotent cell-types (i.e. hematopoietic stem cells) and mesenchymal differentiated cell-types (i.e. fibroblasts) were also analysed (data not shown). The analysis of these samples in an unbiased manner showed that all the mesenchymal SCs team up together with a quite clear separation from the hematopoietic SCs according to their global transcriptomic profiles (Additional file 8: Figure S4). This analysis also indicated that differentiated fibroblasts (FIBs) are much closer to MSCs than to HSCs, but they are also well separated from the mesenchymal lineage. In conclusion, we observed that the MSCs from BM and PL origin have a close expression pattern in full agreement with the RNA-Seq results and with a clear distance from the bone marrow hematopoietic stem cell linage.

### Exploring for functional categories in the MSC transcriptome

**Figure 4:**
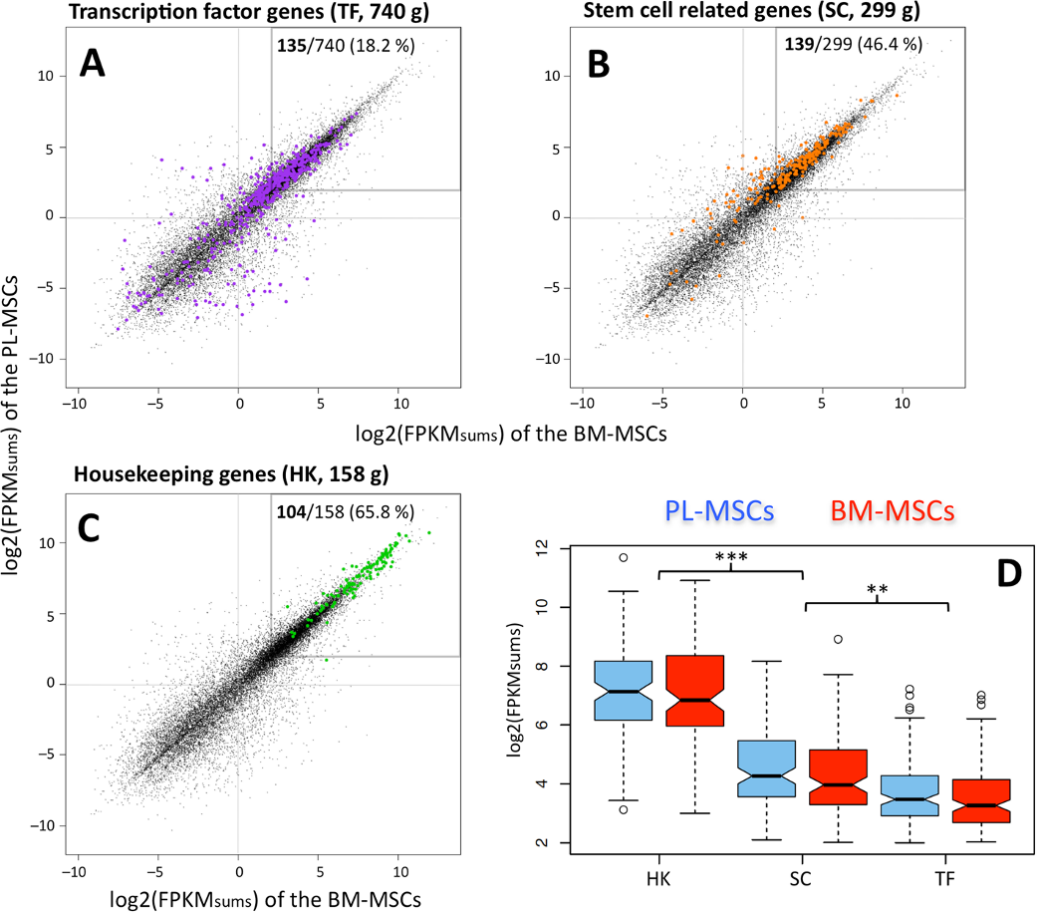
**Mapping the expression of several gene-sets on RNA-Seq data of MSCs.** Scatter plots of *log2 (FPKM*_*sum*_ *)* in BM- against PL-MSC samples marking in colors different groups of genes: **(A)** set of human transcription factors (TF) including 740 genes (purple dots), with135 genes found in the MSC expressed region (hypergeometric *p-value* > 0.95, not significant); **(B)** set of 299 stem cell (SC) related genes (orange dots), including 139 found in the MSC expressed region (hypergeometric *p-value* = 5.96x10^-17^, show significant enrichment); **(C)** set of 158 highly conserved housekeeping (HK) genes (green dots), including 104 found in the MSC expressed region (hypergeometric *p-value* = 1.31x10^-28^, show significant enrichment). **(D)** Boxplot of the *log2(FPKM*_*sum*_ *)* expression distributions of the 3 gene sets (HK, SC, TF) corresponding to PL-MSCs (blue) or BM-MSCs (red).

Digging deeper into this transcriptional profile, several gene-sets involved in distinct biological functional categories were analysed. Looking for specificity of function, a reference set of 740 human TFs was studied, discovering 135 of them in the MSC footprint (Figure 4A; listed at Additional file 9: Table S3). These factors would be the specific regulators of MSCs, which conserve and maintain their cellular characteristics. As expected, this subset does not represent significant enrichment since it includes only 18.2% of known human TFs. A more specific set of 299 human genes associated to stem cell (SC) function was also mapped to the MSC genes, finding 139 positives. This showed a significant enrichment (46.4%) and these genes occupied more extensively the box that included the MSC expression distribution (Figure 4B). Genes included in this stem cell signature are involved in cell cycle regulation, DNA repair, or apoptosis control, as part of the machinery associated to self-renewal. Finally, considering the MSC as a cellular entity, the mapping to a set of well-known cellular housekeeping genes (HK) exhibited an expected strongest presence at the highest expression levels (Figure 4C).

Comparison of the expression distributions obtained for the three analysed gene functional categories (HK, SC, TF) (Figure 4D) showed significant differences: *p-value* [HK] *versus* [SC & TF] <10^−15^; *p-value* [SC] *versus* [TF] <10^−6^ (all parameters of the statistical tests presented in this figure are included in Additional file 10: Table S7). This analysis disclosed a trend of lower expression levels associated to more specific genes (SC and TF) and higher expression to more general genes (HK) common to many different cell types. This observation has strong biological significance but, as far as we know, it has not been reported very often in transcriptomic studies [49]. Finally, a functional enrichment analysis on KEGG signaling pathways was performed for the 5,271 MSC gene dataset versus the complete human genome. Details of annotated terms and p-values are presented in Table 1. This analysis showed high enrichment in the mTOR signaling pathway, as well as in ERBB, TGFβ, NOTCH and WNT pathways. Enrichment in the osteoclast differentiation pathway was also detected. Several of these pathways play important roles in the regulation of cell growth and proliferation, cell survival and differentiation. For example, dysregulated mTOR signaling fuels the destructive growth of cancers [50] and it has been shown that mTOR is essential for growth and proliferation in early mouse embryos and embryonic stem cells [51]. Moreover, accurate tuning of mTOR and WNT pathways have been related to self-renewal, that is the process by which stem cells divide to make more stem cells, perpetuating the stem cell pool throughout life [52]. Maintenance of a stem cell pool requires a finely tuned balance between self-renewal and differentiation and mTOR pathway plays a key role in this regulation [53].

**Table 1:** **Functional enrichment on KEGG signaling pathways of MSCs expressed coding genes**

KEGG pathway	Gene set size	Observed hits	Adjusted p-value*	Hits (Gene_Symbols)
mTOR signaling pathway (hsa04150)	52	27	0.0000505	AKT1, AKT3, CAB39, CAB39L, EIF4E2, EIF4EBP1, MAPK1M MAPK3, MLST8, MTOR, PIK3CB, PIK3R2, PRKAA1, PRKAA1, RHEB, RPS6, RPS6KA2, RPS6KA3, RPS6KB1, RPS6KB2, STK11, TSC1, TSC2, ULK1, ULK3, VEGFA, VEGFB, VEGFC
ErbB signaling pathway (hsa04012)	87	35	0.0019942	ABL1, AKT1, AKT3, BAD, CDKN1A, CDKN1B, CRK, CRKL, EGFR, EIF4EBP1, ELK1, ERBB2, GRN2, GSK3B, HBEGF, HRAS, KRAS, MAP2K1, MAP2K2, MAP2K4, MAP2K7, MAPK1, MAPK3, MAPK8, MAPK9, MTOR, MYC, NCK2, NRAS, PAK1, PAK2, PIK3CB, PIK3RS, RPS6KB1, RPS6KB2
TGF-beta signaling pathway (hsa04330)	83	33	0.0032629	BMPR1A, BMPR2, CDKN2B, CUL1, E2F4, ID1, ID3, MAPK1, MAPK3, MYC, PPP2CB, PPP2R1A, PPP2R1B, RBL2, RBX1, ROCK1, ROCK2, RPS6KB1, RPS6KB2, SKP1, SMADA, SMAD4, SMAD7, SMURF2, SP1, TFDP1, TGFB2, TGFBR1, TGFBR2, THBS2, ZFYVE16, ZFYVE9
Notch signaling pathway (hsa04330)	46	20	0.0056174	ADAM17, APH1A, CIR1, CTBP1, CTBP1, CTBP2, DTX3, DTX3, DTX3L, DVL1, DVL2, HDAC1, HDAC1, HDAC2, HES1, NCOR2, NOTCH2, NOTCH3, NUMB, NUMBL, PSEN2, RBPJ, RFNG
Osteociast differentiation (hsa04380)	127	45	0.0088651	AKT1, AKT3, CHUK, CYBA, FHL2, FOS, FOSB, FOSB, FOSL1, FOSL2, GRB2, IFNAR1, IFNAR2, IFNGR1, IFNGR2, IFNGR2, IKBKB, IRF9, JAK1, JUNB, JUND, MAP2K1, MAP2K7, MAP2K7, MAP3K7, MAPK1, MAPK12, MAPK14, MAPK3, MAPK3, MAPK8, MAPK9, NFKBIA, PIK3CB, PIK3R2, PP3CA, PPP3CC, RAC1, RELA, SIRPA, SOCS3, SQSTM1, STAT2, TAB2, TGFB2, TGFBRI, TGFBR2, TNFRSF11B, TYK2
Wnt signaling pathway (hsa04310)	150	51	0.0134525	CCND1, CSNK2A1, CSNK2A2, CSNK2B, CTBP1, CTBP2, CTNNB1, CTNNBIP1, CUL1, DVL1, DVL2, FOSL1, FZD2, FZD4, FZD6, FZD7, FZD8, GSK3B, LRP5, LRP6, MAP3K7, MAPK8, MAPK9, MYC, PLCB3, PPP2R5C, PPP2RSE, PPP3CA, PPP3CC, PPP3R1, PRICKLE1, SENP2, SFRP4, SIAH1, SKP1, SMAD2, SMAD4, TCF7L1, WNT5B
Apoptosis (hsa04210)	87	32	0.0136314	AIFM1, AKT1, AKT3, BAD, BAX, BCL2L1, BID, BIRC2, CAPN1, CAPN2, CASP6, CASP8, CAPSP9, CFLAR, CHUK, ENDOG, FADD, IKBKB, IRAK1, NFKBIA, PIK3CB, PIK3R2, PPP3CA, PPP3CC, PPP3R1, PRKACA, PRKAR1A, RELA, RIPK1, TNFRSF10D, TRADD, XDIAP
VEGF signaling pathway (hsa04370)	75	28	0.0160140	AKT1, AKT3, BAD, CASP9, CDC42, HRAS, HSPB1, KRAS,
Adipocytokine signaling pathway (hsa04920)	67	25	0.0207573	ACSL1, ACSL3, ADIPOR1, ADIPOR2, AKT1, AKT3, CHUK, CPT1A, IKBKB, MAPK8, MAPK9, MAPK9, MTOR, NFKBIA, NFKBIB, PCK2, PRKAA1, PRKAB1, PRLAB2, PRKAG1, PTPN11, RELA, SOCS3, STAT3, STK11, TRADD

*Hypergeometric test (*p-values* adjusted using Benjamini and Hockberg method; done with *HTSanalyze* R-pakage).

### Meta-regulators: master controllers of the MSC transcriptome

Transcription factors are key regulators of cell fate decisions, carrying out the modulation of the expression flow. Among the MSC expressed genes, 135 were found to be TFs according to the mapping over a census of 740 human TFs [27, 28] (see Methods). To derive which of them have a broader spectrum of action we explored the promoter regions of the 5,271 genes that constitute the MSC gene expression footprint, searching for binding sites of the 135 TFs through their *cis*-regulatory regions of the whole MSC genes. To do it we performed an enrichment analysis over detected TF-binding-sites (TFBSs) in the DNA sequences upstream of the 5,271 genes. Only TFBSs recognized by the 135 MSC-TFs were considered. Bioinformatic tools TransFind [34] and oPOSSUM [35] were used, applying two alternative TFBS-motif databases: JASPAR Core from mammals [30] and TRANSFAC from primate orthologous [31]. Different tools provide different analytic algorithms for individual motif detection, affinity binding, matrix scoring, and statistical testing of over-representation (see Methods). For this reason we apply several methodologies in the search for TFBSs. The results of these analyses found several TFBS_matrices enriched with quite significant values (Additional file 1: Table S4A) that allow to identify the corresponding TFs; for example, matrices MA0004.1 and MA0006.1 corresponded to factor ARNT. A set of 17 statistically significant TFs was identified: ARNT, ATF4, CREB3, EGR1, ELK1, ETS1, HES1, KLF4, MAX, MYC, NFYA, NFYB, NFYC, SP1, USF1, USF2 and VDR. Each TF was found in at least two of the three searches done (see Additional file 1: Table S4A).

**Figure 5:**
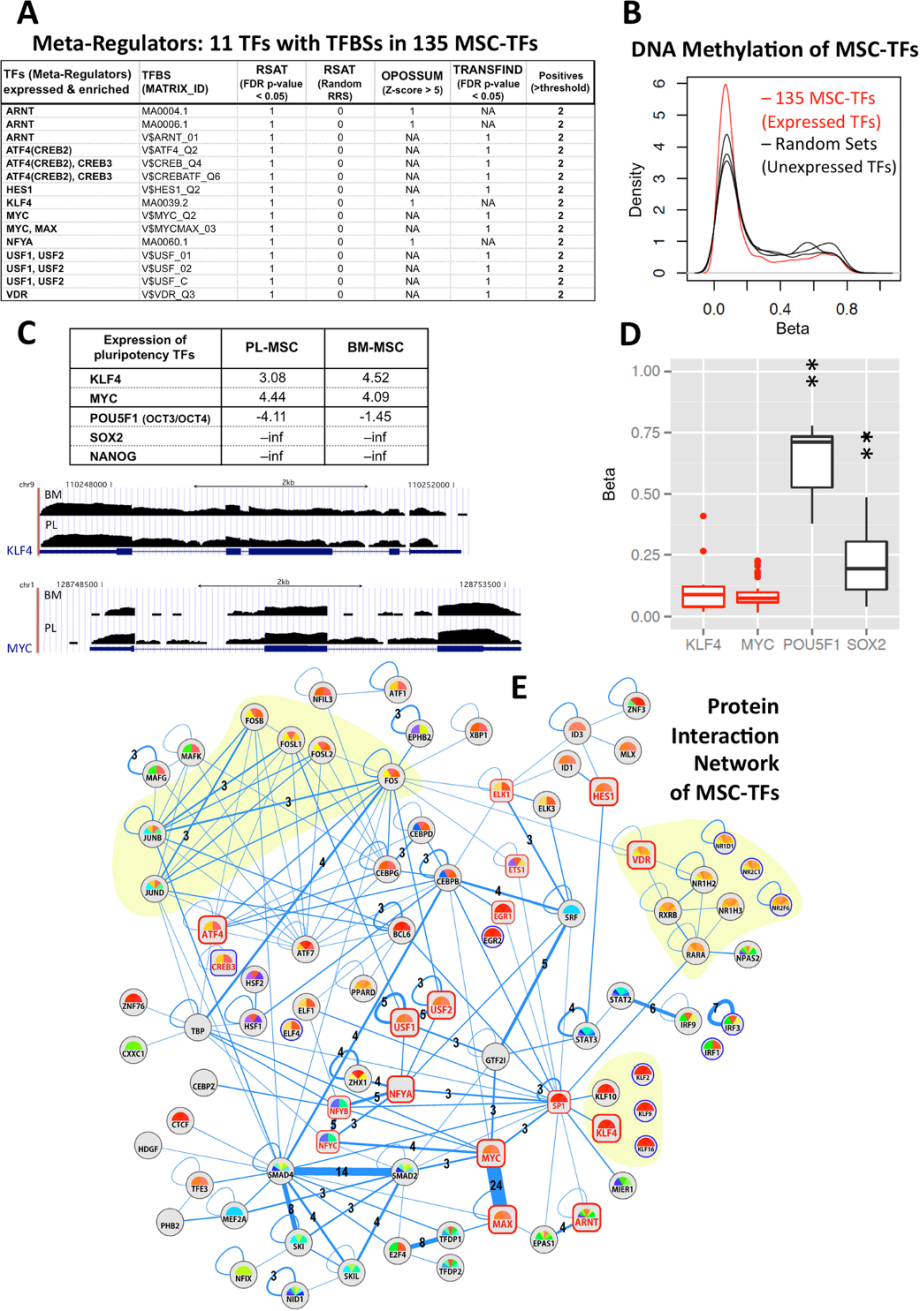
**Transcription factors within the MSC expression footprint. (A)** Table of 11 meta-regulators found to be enriched on the TFBS of 135 TFs detected in the MSC expression footprint. **(B)** DNA methylation distributions –densities versus Beta values– corresponding to 135 expressed MSC-TFs (red) and to other sets of 135 randomly selected TFs (black). The plot shows higher accumulation of methylation measurements around lower values of Beta for MSC-TFs. **(C)** Table of expression values of *log2 (FPKM*_*sum*_ *)* corresponding to five TFs related to pluripotency (KLF4, c-MYC, POU5F1, SOX2 and NANOG); and RNA-Seq raw profiles of KLF4 and MYC genes in BM- and PL-MSC samples. **(D)** DNA methylation distributions –Beta values– corresponding to the CpGs associated to 4 TFs. Wilcoxon tests proving significant differences in these analyses gave the following p-values: KLF4 *vs* POU5F1, p-value = 2.40×10e^-4^; KLF4 *vs* SOX2, p-value = 2.63×10e^-2^; MYC *vs* POU5F1, p-value = 1.89×10e^-6^; MYC *vs* SOX2, p-value = 0.782×10e^-6^ (all parameters of the statistical tests presented in this figure are included in Additional file 12: Table S8) **(E)** Protein interaction network including the MSC-TFs found. Edge-thickness (**blue** lines) and number represents the number of experimental evidences that support a given protein-protein interaction (PPI). Shaded groups represent structural families. The 17 TFs that were found to regulate the MSCs gene expression footprint are labeled with **red** names and enclosed by a **square** (instead of a **circle** like the rest of the nodes). Within these 17 TFs, the nodes corresponding to the 11 TF meta-regulators (detailed in **5A**) are also labeled with **red** names but larger **squares**. Nodes with border in **blue** (11 genes) are not linked in the network, but they are structural paralogs of some of the linked nodes placed aside.

Going further, we investigated if those 17 TFs could be broad regulators of the TFs alone. In this way, a second query searched for the TFBSs enriched in the set of 135 MSC-TF genes. Since this set is small, an additional tool (*RSAT*[33]) was applied. *RSAT* allowed using a random reference set (RRS) as a negative control in order to penalize false positive enriched motifs. Over-represented position matrices found with at least 2 of the 3 methods applied, were associated with their corresponding TFs (Additional file 1: Table S4B). Among the list of 17 TFs, 11 were found significantly enriched in the 135 MSC TF set (summarized in Figure 5A). Since these TFs can be considered that regulate the regulators, we refer to them as “meta-regulators”. Two of these depicted meta-regulators, KLF4 and MYC, are included in the well-known set of induced pluripotent stem cell (iPSC) factors (i.e. the Yamanaka factors) [54], but the other two iPSC factors (POU5F1 and SOX2) were found not expressed in the MSCs (expression shown at Figure 5). Other TFs that have been identified as regulators of embryonic stem cells (ESCs), like homeoprotein NANOG, were again not detectable in the MSCs. Together with factors specific of stem cells, the set of 11 meta-regulators also included some pleiotropic TFs constitutively active in eukaryotic cells, like ARNT [49].

### DNA methylation state of TFs

To provide additional data supporting the validity of the proposed MSC transcriptional portrait, methylation levels of CpG sites mapping the 135 MSC expressed TFs were studied. To do this, a set of five methylation arrays of normal human MSCs from bone marrow donors [38] were examined. Beta values of CpGs annotated to MSC-TF genes showed a clear hypomethylation pattern (Figure 5B). In fact, when comparing with the methylation corresponding to sets of any 135 human TFs randomly selected, we observed that most of the CpGs had lower Beta levels in the MSC-TFs (see density curves in Figure 5B). This observation is repeated when the CpGs are redistributed by the island sub-regions (i.e. in islands, shelves and shores, north or south positioned, obtained according to [40], see Additional file 11: Figure S3). All these sub-regions appear hypomethylated in the MSC-TF set, the islands and the north-shores appearing as the most clearly hypomethylated regions. Following these results, epigenetic hypomethylation over the meta-regulators was verified too. KLF4 and MYC were significantly hypomethylated compared to the not expressing POU5F1 and SOX2 (Figure 5D and Additional file 12: Table S8).

### TFs interaction network

Transcriptional gene regulation in human cells is not individually controlled. TFs are DNA-binding proteins that act coordinately to activate or repress gene transcription. To illustrate possible associations or links expected to occur between the 135 TFs that we have detected in the MSC transcriptomic profile, we built a TF-network of relations based on reported protein-protein interactions (PPIs) that have experimental evidences of physical interaction [36]. Interactions among 135 MSC-TFs gave out a network that included 74 nodes connected through 197 edges (Figure 5E) (in this figure another 11 not-connected nodes were added because they are paralogos of some connected node). Some TF families resulted well represented: the FOS-JUN family with many PPI links, the VDR-NR family (nuclear receptors C4 zinc-fingers) and the KLF family (all enhanced with yellow background in the figure). Joint regulation activity can be expected by the physical binding of well reported interactions such as MYC-MAX and SMAD2-SMAD4; but other interesting interactions and interaction groups were revealed by the network: SMAD2-SKIL and SMAD4-SKI; E2F4-TFDP1; STAT2-IRF9; NFYA-NFYB-NFYC; USF1-USF2; SRF-GTF2I and SRF-CEBPB. Sounds plausible that these protein pairs work together in regulatory maintenance of the MSC, but further experimental studies should be done to determine in which specific context they react and how they contribute to the system.

### Placenta versus bone marrow MSCs differential expression

**Figure 6:**
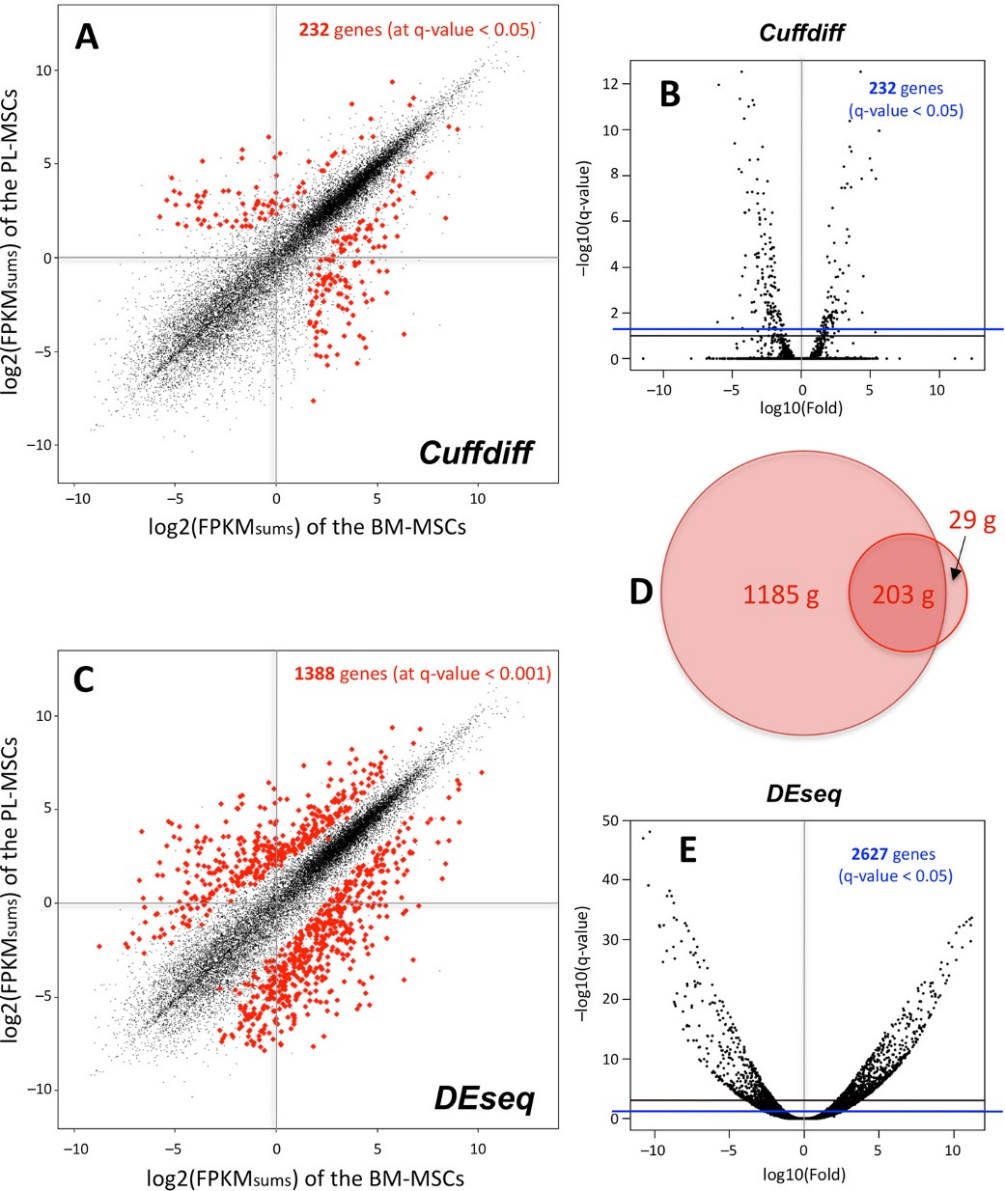
**Analysis of differential expression on the RNA-Seq data of BM- and PL-MSCs.** Scatter plots marking in red the significant genes found with two different methods: **(A)** *Cuffdiff* and **(C)** *DEseq*. Volcano plots of the expression data analyses done **(B)** using *Cuffdiff* and **(E)** using *DEseq*. Setting the *q-value* threshold at < 0.05 (blue line) for both methods, they detect 232 and 2627 significant genes, respectively. Since *DEseq* method is much less stringent than *Cuffdiff* and to avoid false positives, a second cut-off at *q-value* < 0.001 was set up for *DEseq* differential expression, selecting in this way 1388 significant genes. **(D)** Figure showing a proportional Venn diagram to illustrate the overlap of the genes selected by *Cuffdiff* (232) and *DEseq* (1388). The overlap includes 203 genes that undergo significant differential expression changes (i.e. common genes in red, in the scatter plots **6A** and **6C**).

Within the scatter plots presented in Figure 3A, dispersed gene dots separated from the diagonal can be observed. In order to investigate the differences that these more variable genes may entail and find significant differentially expressed (DE) genes, we applied two algorithms: *DEseq* and *Cuffdiff* (Figure 6). Scatter plots showing the significant DE genes (Figure 6A,C) and volcano plots (Figure 6B,E) indicated that different algorithms provided different results. Setting the q-value threshold at < 0.05 for both methods, they detected 2,627 and 232 significant genes respectively. This indicated that *DEseq* method is much less stringent than *Cuffdiff*. To restrict the number of false positives, we moved the cut-off for *DEseq* to q-value < 0.001 (that gave then 1,388 genes) and searched for the genes that were significant in both methods. The overlapping genes were extracted, obtaining a set of 203 (which corresponded to 87.5% of the *Cuffdiff* result) (Figure 6D). Within these genes and according to *Cuffdiff*, 125 were up-regulated in BM-MSCs and 78 up-regulated in PL-MSCs (Additional file 13: Table S5). In 14 of these genes we denoted a disagreement in the direction of the differential change reported by *Cuffdiff* and *DEseq* (underlined in Additional file 13: Table S5). Despite this, we trust the result of *Cuffdiff* and so we analyzed the set of 203 genes. This set provides a fair measure of the distance between the two types of MSCs, representing the 4% of the common gene profile described above (203 over 5,271 genes). The study of this set to extract biological meaning using functional enrichment analyses (Additional file 14: Table S6), indicated that BM-MSC genes were enriched in functions such as: bone biogenesis, bone formation, blood vessel morphogenesis, extracellular matrix organization and inflammatory response; which comprise programs underlying the specific role of the MSCs in the bone marrow microenvironment with features ligated to hematopoietic regulation [55, 56]. By contrast, PL-MSC genes pointed towards specific terms much linked to the stem cell nature, such as: embryonic morphogenesis, cell cycle activation and negative regulation of cell death; which are in agreement with the fetal origin and with the observed rapid growth capacity of this mesenchymal cell subtype.

To complete the analysis of the differences between BM- and PL-MSCs we looked for differential splicing events between both sample types. *Cuffdiff* algorithm allows identifying not only genes, but also isoforms that show significant differential expression between two sample sets. This comparison yielded 297 genes for which distinct isoforms showed differential expression between the BM- and the PL- samples; but only 16 of these genes were included in the common transcriptomic footprint of 5,271 genes (i.e. only 0.3% of the MSC genes, expressed different isoforms in BM *versus* PL). By contrast, 141 of the 297 genes that suffered alternative splicing were included in the signature of 203 genes that was reported as differential expression between BM- and PL-MSCs (i.e. a 69.4% of the genes that differentiate both subtypes correspond also to different isoforms in BM *versus* PL). In conclusion, these results corroborate the biological expectation that same cell-types (MSCs) with different tissue origin (BM- *versus* PL-) would have a main large common gene expression footprint with a small differential gene expression signature, and –in such differential signature– most of the genes would be different because they present alternative spliced isoforms.

## Discussion

### Human MSCs from bone marrow and placenta

An increasing number of publications backup the properties of MSCs from bone marrow. In fact, the BM-MSCs are considered the milestone of the MSC definition. PL-MSCs have been described more recently, and slight efforts have been focused on proving their abilities [13]. In this work we have shown their faster growth on plastic, their differentiation capacity *in vitro* towards reference lineages and their exposed characteristic immunophenotype. Combined data from separated environments led to capture of the common features of MSCs, as much as the dissimilarities were delineated by the tissue-associated backgrounds. A list of 203 differentially expressed genes was found, that translates into about 5% distance between BM-MSCs and PL-MSCs lineages. A study of murine bone marrow MSCs, comparing their gene expression profiles with brain and muscle MSCs, yielded 197 and 125 DE-genes respectively (considering expression differences >2.2 fold) [57]. Wagner *et al.* using microarray data [58] reported 478 genes changed when compared human MSCs from three tissue origins (bone marrow, adipose tissue and umbilical cord blood) to differentiated fibroblasts. Our overall common profile indicates too that the MSC subtypes studied are biologically close, regardless of being located far away from each other in time and space.

Another expression comparative work disclosed a specific list of genes up-regulated in BM-MSCs [59]. Several of these genes (HLA-DRB1, ENPP1, KCNN4 and EN1) were also found in our set of 125 genes significantly over-expressed in BM-MSCs. ENPP1 has been associated to calcium deposition disorders [60], KCNN4 to chondral ossification [61], and Engrailed (EN1) is a homeobox containing TF, regulator of growth and development processes, among them, ossification [62]. When our set of BM up-regulated genes were submitted to functional enrichment analysis, again bone related annotations came out. These findings highlight genes of the long reported role of BM-MSCs over other tissue origins in bone homeostasis and regeneration.

### The MSC gene expression profile

Following RNA-Seq data analyses, a common gene expression footprint to BM- and PL-MSCs was depicted. Analyses of human cell-specific transcriptomes using RNA-Seq provide reliable results. Recent studies on a model human cell line (HeLa cells) have shown that deep transcriptome and proteome mappings done in parallel with RNA-Seq and with advanced mass spectrometry (MS)-based proteomics provide quite coherent and consistent results of a single cell type [63]. The genome-wide expression portrait of MSCs that we presented in this work comprises the largest non-relative profile released for this human primary cell type. In Tsai *et al.*[59], 47 genes were found to be a specific MSC core gene signature when contrasting microarray data from four human perinatal tissues against a mixture of differentiated cells. From of this small signature, 31 genes (66%) were present among the transcriptome of MSCs. Using a similar approximation, Pedemonte *et al*. [64] identified in mice a specific molecular signature of MSC in the hematopoietic niche enclosing 381 genes, showing for example enrichment in WNT pathway genes that we also observed enriched in our global profile. In general, as far as we can observe, all the signatures reported for MSCs have been primarily derived from comparative measures of differential expression (i.e. they are relative measurements) and there is not a reported full profile of the genes expressed in human MSCs.

### Signaling pathways enriched in the MSCs

Analysing the 5,271 MSC genes and the pathways they are involved in, vast cellular processes become evident. Some enrichment on signaling pathways can reveal key integrators that coordinately drive toward cell decisions. Globally, TGF-β, mTOR or WNT pathways stay behind “cell quiescence”, “self-renewal”, “maintenance”, “growth and apoptosis control”, as well as “differentiation” and “reprogramming”. These pathways are also quite relevant in cancer and metastasis, establishing links between self-renewal cells and cancer cells. The relationship between these pathways and their effect on the processes of epithelial to mesenchymal transition (EMT) might also be of interest. TGF-β is a potent inducer of EMT that activates key regulators such as SNAI1/2, TWIST and ZEB1/2 [65]. It is noteworthy that SNAI2 and TWIST1 exhibit active expression in our cells. In Gulhati *et al.*[66], establishment of metastasis through EMT of colon cancer cells was completely abolished upon inhibition of mTOR. Hedgehog signaling cascade crosstalk with WNT, epithelial/fibroblast growth factors, and TGF-β/Activin/Nodal/BMP signaling cascades, are implicated in EMT through E-cadherin repression [67, 68]. All these routes and central genes appear highly enriched in our MSC footprint, thus establishing identity connections between the “mesenchymalized” epithelial cells, and the mesenchymal phenotype itself.

### Candidate markers for MSCs

Several gene products postulated as markers of MSC subpopulations have been surveyed in this work. CD146 (MCAM) was detected in mesenchymal osteoprogenitors that mainly sub-localize in the vascular niches of the bone marrow [44]. Its expression was reported to increase during *in vitro* normoxic culturing [45]. Likely, our BM stromal cultures, as much as the PL ones, strongly transcribe the MCAM gene. The lack of variable levels of FPKMs also denotes homogeneity of expression along the populations. In the case of Nestin gene [42], strikingly increased transcription in PL with respect to BM might be associated to the immaturity stage inherent to a fetal tissue. Nestin is an intermediate filament protein involved in axon guidance of neural progenitors. Recently, this protein has been associated to a small BM subpopulation of MSCs in mice, that is thought to derive from neuro-ectoderm and to be self-renewing sphere-clonogenic cells [43]. MSCs coming from tissues in development might be retaining a greater expression of Nestin or, sidewise, the Nestin expressing population is prevalent in human PL with respect to BM. Nevertheless, to our best knowledge, Nestin expression in human placental mesenchymal cells had not been reported before. Another marker, CD271, have been used to enrich the mesenchymal fraction of BM extracts [47]. This nerve growth factor receptor is again involved in neural survival and differentiation, although its function is not well understood yet. CD271 was not detectable in PL-MSC cultures, and very weak in BM-MSCs. We may attribute this result to the lost of stimuli that cells undergo during *in vitro* expansion, where microenvironment niche signaling is inherently diminished. Lost of CD271 in MSCs throughout passaging has been already described [46].

### Transcription factors of the MSC biology

Two expressed TFs, MYC and KLF4, are pluripotency inductors that exert transcriptional regulation over many genes. Interestingly, they have been denoted by their potential control over mesenchymal differentiation [69] and, moreover, Kruppel-like factor 4 (KLF4) DNA-binding protein has been shown to present a central role in regulating MSC transcriptional activity to maintain cells in an undifferentiated state (i.e. for stemness maintenance) [70]. In fact, KLF4 and other members of this family (KLF2 and KLF9, also expressed in MSCs) showed down-regulation when BM-MSCs (and also MSCs derived from adipose tissue) were submitted to differentiation pressure [56]. Even more, ChIP experiments verified the binding of KLF4 to known active MSC genes; and silencing of KLF4 also provoked their down-regulation. Therefore, KLF4 seems to be a key regulator and maintainer of the MSCs status. Other TFs detected in the MSCs expression footprint seem to be preserved during differentiation process. For example c-MYC, since it has been observed that the binding sites of this TF are highly present in osteogenic genes and differentiation to osteoblast occurs under the over-expression of c-MYC [55].

The potential role of epigenetics on the multipotent cell differentiation capacity of MSCs has been recently studied by Yannarelli *et al.*[71]. These authors showed that the pluripotency factors OCT4 and SOX2 had a very low expression level in BM-MSCs, and they prove methylation of OCT4 in these cells. Our results confirmed the methylation of OCT4 in MSCs (Figure 1D), revealing also the methylation of SOX2 and the hypomethylation of two other key pluripotency factors: KLF4 and c-MYC. The epigenetic status of these later factors corroborates their role in the regulation of MSCs fate.

The analyses of expression regulatory genomic regions on MSCs allowed us to propose that 11 out of 135 expressed TFs might be upper determinants of the MSC expression footprint. However, TFs do not act alone, since they usually form complexes to bind DNA regulatory regions promoting activation or repression of the gene transcription. To investigate which MSC regulators may work together, we built an interaction network that illustrates the wiring between individual TFs. Several enhanced node connections observed in this network correspond to well-reported interactors. For example, many experimental data support the presence of c-MYC–MAX heterodimer, and the increase in this dimer plays a fundamental role in regulating cell cycle entry and proliferation [72]. USF1–USF2 dimer largely regulates genes of fatty acid metabolism [73], and we may speculate their enrolment in MSC differentiation towards the adipogenic phenotype. The Kruppel-like factor family (KLFs) is implicated in a wide range of cellular processes, including proliferation, apoptosis, differentiation, inflammation, migration, tumorigenesis [74]. Interestingly KLF4, as indicated above, is implicated in maintaining stem cell pluripotency and has been reported to perform a cooperative activation with MEIS2 and PBX1 [75] resulting in the fine-tuning of the KLF4 response. Other members of these TFs families, MEIS3 and PBX2, are indeed present in the compendium of MSC TFs here reported, and so they can be postulated as direct interactors of KLF4 in MSCs.

Another interesting module found in the TF-TF network is SMAD4-SMAD2-SKI-SKIL. SKI/SKIL are oncogene homologs involved in TGF-β signaling. When SMAD2 is activated by TGF-β receptor, it dimerizes with SMAD4. The resulting complex recruits in-cell available partner molecules which will determine which gene-sets to activate or repress [76]. Several studies with progenitor cells have presented those partners as lineage specific factors. For example, the human SKI-like (SKIL) gene encodes the SMAD transcriptional corepressor SNON that antagonizes TGF-β signaling and suppresses maturation of chondrocytes by mediating signal cross-talk between TGF-β and bone morphogenetic protein (BMP) pathways [77]. In this way, SKIL overexpression can be another responsible for preventing differentiation of MSCs. A role in self-renewal and differentiation standby is congruent with the oncogenic capacity that has been attributed to SKIL protein in certain conditions [78]. Finally, NFYA-NFYB-NFYC trimer can be a key factor of the MSC expression profile since its regulatory subunit (NFYA) has been shown to activate multiple hematopoietic stem cell (HSC) regulatory genes and to promote self-renewal in these cells [79]. Considering all these facts and reported observations, the envisioned TF-TF network is a feasible map of the interaction and mutual coordination of mesenchymal-type specific gene regulators that helps to define in a comprehensive way the TFs acting in the human MSC transcriptome.

## Conclusions

MSCs are able to regenerate mesoderm-derived tissues in adult organs. Their plasticity and immunomodulatory properties have contributed to their widespread trial in cell therapy biomedical programs over the last few years, however the molecular machinery that defines and channels their behavior still remains poorly understood. Moreover, there are many examples in which new knowledge about cell therapy can only be learned by using direct data from human cells, and tests or trials on model organisms (such as mouse or rat) can not elucidate the specific molecular signature of human cells [80]. As was the case for hematopoietic stem cells in the 1970s and 1980s, the first “in-man” testing with human MSCs in the 1990s and 2000s has been invaluable. No prospective *in vitro* study or animal testing could provide the knowledge attained through such actual human exposure [80]. These arguments show clear the substantial value of achieving an adequate molecular cartography of the human MSCs. The work here presented gives a significant step in this direction providing the first complete view of the expressed transcriptome of this specific human cell type isolated from two quite distinct tissue microenvironments: adult bone marrow and fetal placenta.

Considering different tissue origins, the multipotential capacity of both subtypes of human mesenchymal cells (BM- and PL-MSCs) was confirmed and immuno-phenotyping provided verification of the cells population homogeneity. In this way, the RNA deep sequencing assays here presented were performed on well-controlled human cell populations, and allowed quantitative determination of a human MSCs genome-wide expression portrait that includes a compendium of 5,271 protein-coding genes. This valuable resource confirmed the expression of all the known CD markers expected in MSCs and revealed some other expressed markers –such as MCAM and NES– that are still controversial in some forums. A set of TFs activated in MSCs was also identified, revealing the presence of meta-regulators like KLF4 that has been implicated in self-renewal processes. mTOR pathway was also found as highly activated in the functional enrichment analysis of the MSCs transcriptome, and mTOR is directly implicated in the fine tuning between self-renewal quiescence and differentiation that any SCs population needs. The functional analysis of the MSC expression footprint also showed the presence of other important regulatory gene-sets (such as pluripotency associated genes) and the enrichment on signaling pathways (such as TGFβ and WNT pathways). In this way, the work provides a newly determined gene active portrait of human MSCs that delineates the molecular nature of this cell population. This portrait can be very helpful for comparisons with the transcriptomic footprint of other human cell types and stem cell lineages.

Finally, as far as we know, all the expression signatures so far reported for human MSCs are derived from relative measures and differential analyses, and therefore, our work comprises a non-relative approach to determine the transcriptome of human primary MSCs trying to answer a simple but critical question: what genes are expressed active in this cell type at its ground self-renewal state?

## Electronic supplementary material

Additional file 1: Table S4.: File including two worksheets listing the TFBSs enriched **(A)** in the 5,271 genes of the MSC expressed signature and **(B)** in 135 genes of the MSC-TFs signature. Specific parameters (i.e. Z-score, FDR p-value) related to each run of the TF set analysis are detailed in the tables. (XLS 26 KB)

Additional file 2: Figure S1: Microscope photos of MSCs differentiation assays to osteoblasts, adipoblasts and chondroblasts. Multipotent *in vitro* differentiation assays performed with samples of BM-MSCs (blue labels) and PL-MSCs (red labels). Left-handed photos show MSCs passed through differentiation induction (i.e. positive assays). Negative controls are shown in the right hand photos. **(A)** Osteogenic differentiation detected by alkaline phosphatase (AP) activity. Arrows indicates pools of high of AP activity inside the cells. **(B)** Adipogenic differentiation detected by fat staining with Oil-Red-O. Arrows point out fat vacuoles stained in red inside the cell cytoplasms. **(C)** Chondrogenic differentiation detected by tissue three-dimensional growth. Images show the section of cartilage spheroids stained with Hematoxilin-Eosin. Arrows denote areas of matrix composition produced by cells embedded into it. (ZIP 1 MB)

Additional file 3: Figure S2: Scheme describing of the design and outcome of the RNA sequencing process. **(A)** Samples input: 3 biological replicates of each sample type, that were splitted in two technical replicates (only 3 biological replicates of each type were fully sequenced). **(B)** Sequencing and alignment details. **(C)** Table showing the number of reads obtained for each sample and the results of the mapping to human gene loci using GSNAP alignment tool. (PNG 73 KB)

Additional file 4: Table S1: List of 8,534 genes expressed in MSCs over the cut-off *log2* (FPKM_sums_) ≥ 2. (XLS 2 MB)

Additional file 5: Table S1b: Tab delimited format corresponding to the list of 8,534 genes expressed in MSCs over the cut-off *log2* (FPKM_sums_) ≥ 2. (TXT 653 KB)

Additional file 6: Table S2: List of 5,271 protein-coding genes of the MSC signature that are expressed in MSCs over the cut-off *log2* (FPKM_sums_) ≥ 2. (XLS 803 KB)

Additional file 7: Table S2b: Tab delimited format corresponding to the list of 5,271 protein-coding genes of the MSC signature that are expressed in MSCs over the cut-off *log2* (FPKM_sums_) ≥ 2. (TXT 426 KB)

Additional file 8: Figure S4: Comparative transcriptomic profiling of human MSCs versus related cell-types using genome-wide expression exon microarrays. MSCs samples isolated from three different tissue origins (bone marrow BM, placenta PL and adipose tissue AD) are compared with hematopoietic stem cells (HSC) and differentiated fibroblasts (FIB). The samples were analyzed using *Affymetrix* Human Exon 1.0 exon arrays, which have coverage for 20,238 unique human gene loci. The full expression signal of the arrays was normalized and calculated with RMA algorithm (using *affy* package from Bioconductor). Unsupervised hierarchical clustering of the global gene expression signatures was done to compare the samples. The heatmap shows the result of such clustering analysis. All genes were used for the distance calculations. The dendrogram of the sample clustering is also shown. Color scale provides a view of the distance range. (PNG 168 KB)

Additional file 9: Table S3: File including worksheets listing three functional gene-sets mapped to the MSC signature (104 HK-genes, 139 SC-genes and 135 TF-genes). (XLS 76 KB)

Additional file 10: Table S7: Table including the data corresponding to the statistical tests performed to compare the gene expression data distributions presented in Figure 4D. (XLS 24 KB)

Additional file 11: Figure S3: Analysis of the methylation data that is described in the manuscript. Boxplot of methylation distributions of the 135 pictured MSC-TFs (in red) compared to a negative set of other 135 TFs (in blue) not present in the MSC footprint. Different regions of the CpG islands were analyzed and shown in this plot. Beta values represent the methylation levels. (PNG 36 KB)

Additional file 12: Table S8: File including the data corresponding to the statistical tests performed to compare the DNA methylation data distributions presented in Figure 5D. (XLS 10 KB)

Additional file 13: Table S5: Table containing the 203 genes differentially expressed between PL-MSC and BM-MSC and all the statistical parameters provided by the two methods (*Cuffdiff* and *DEseq*) applied to detect such genes. (XLS 122 KB)

Additional file 14: Table S6: File including two worksheets with the functional enrichment analyses of the differential genes up-regulated in BM-MSC (125 genes) and up-regulated in PL-MSC (78 genes). (XLS 22 KB)

## Abbreviations

TF: Transcription factor
TFBS: Transcription factor binding site
MSC: Mesenchymal stromal/stem cell
BM: Bone marrow
PL: Placenta
DE: Differential expression.
