# Supplementary figures and images for: Transcriptomic portrait of human Mesenchymal Stromal/Stem cells isolated from bone marrow and placenta
Source: BMC Genomics. 2014 Oct 19;15(1):910. doi: 10.1186/1471-2164-15-910 (PMC4287589; doi:10.1186/1471-2164-15-910)

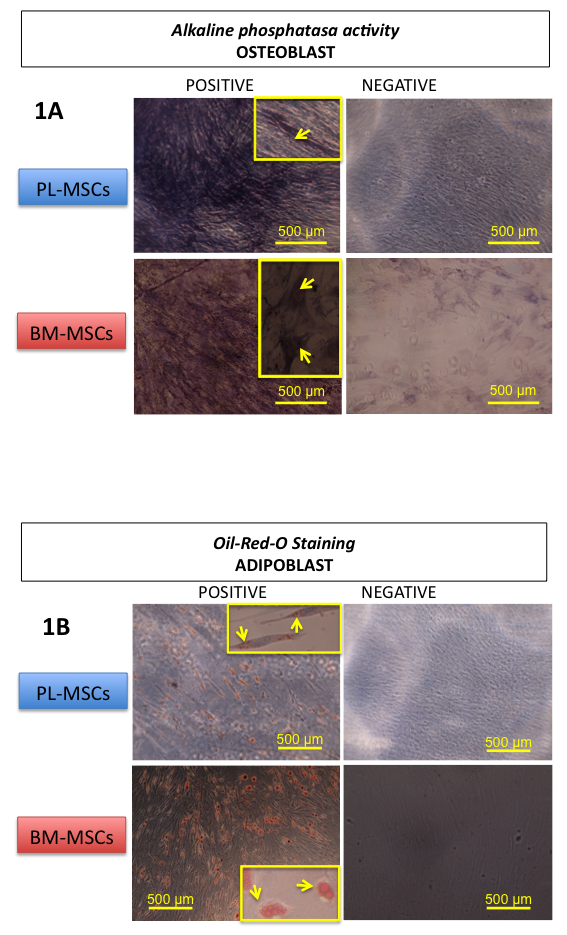

Supplement: Supplementary file 2 — Additional file 2: Figure S1: Microscope photos of MSCs differentiation assays to osteoblasts, adipoblasts and chondroblasts. Multipotent in vitro differentiation assays performed with samples of BM-MSCs (blue labels) and PL-MSCs (red labels). Left-handed photos show MSCs passed through differentiation induction (i.e. positive assays). Negative controls are shown in the right hand photos. (A) Osteogenic differentiation detected by alkaline phosphatase (AP) activity. Arrows indicates pools of high of AP activity inside the cells. (B) Adipogenic differentiation detected by fat staining with Oil-Red-O. Arrows point out fat vacuoles stained in red inside the cell cytoplasms. (C) Chondrogenic differentiation detected by tissue three-dimensional growth. Images show the section of cartilage spheroids stained with Hematoxilin-Eosin. Arrows denote areas of matrix composition produced by cells embedded into it. (ZIP 1 MB) [file 12864_2014_6787_MOESM2_ESM.zip › 3434733931296990_MOESM7_ESM.png]

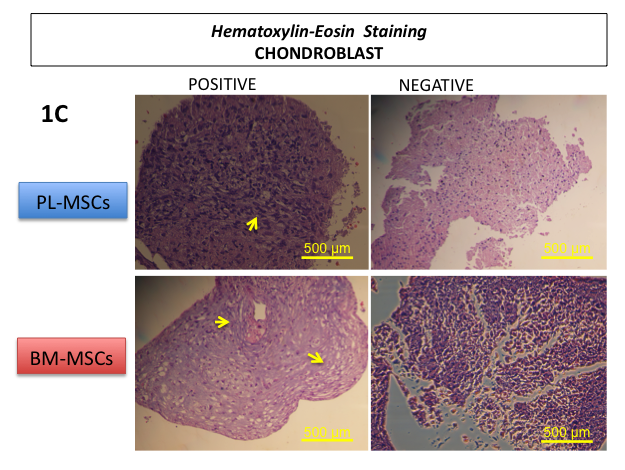

Supplement: Supplementary file 2 — Additional file 2: Figure S1: Microscope photos of MSCs differentiation assays to osteoblasts, adipoblasts and chondroblasts. Multipotent in vitro differentiation assays performed with samples of BM-MSCs (blue labels) and PL-MSCs (red labels). Left-handed photos show MSCs passed through differentiation induction (i.e. positive assays). Negative controls are shown in the right hand photos. (A) Osteogenic differentiation detected by alkaline phosphatase (AP) activity. Arrows indicates pools of high of AP activity inside the cells. (B) Adipogenic differentiation detected by fat staining with Oil-Red-O. Arrows point out fat vacuoles stained in red inside the cell cytoplasms. (C) Chondrogenic differentiation detected by tissue three-dimensional growth. Images show the section of cartilage spheroids stained with Hematoxilin-Eosin. Arrows denote areas of matrix composition produced by cells embedded into it. (ZIP 1 MB) [file 12864_2014_6787_MOESM2_ESM.zip › 3434733931296990_MOESM8_ESM.png]

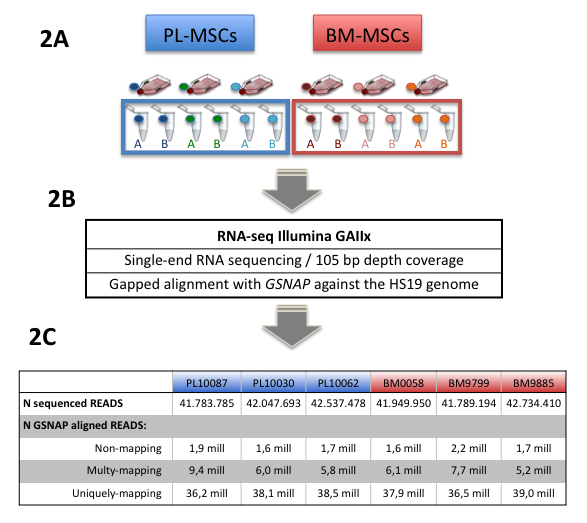

Supplement: Supplementary file 3 — Additional file 3: Figure S2: Scheme describing of the design and outcome of the RNA sequencing process. (A) Samples input: 3 biological replicates of each sample type, that were splitted in two technical replicates (only 3 biological replicates of each type were fully sequenced). (B) Sequencing and alignment details. (C) Table showing the number of reads obtained for each sample and the results of the mapping to human gene loci using GSNAP alignment tool. (PNG 73 KB) [file 12864_2014_6787_MOESM3_ESM.png]

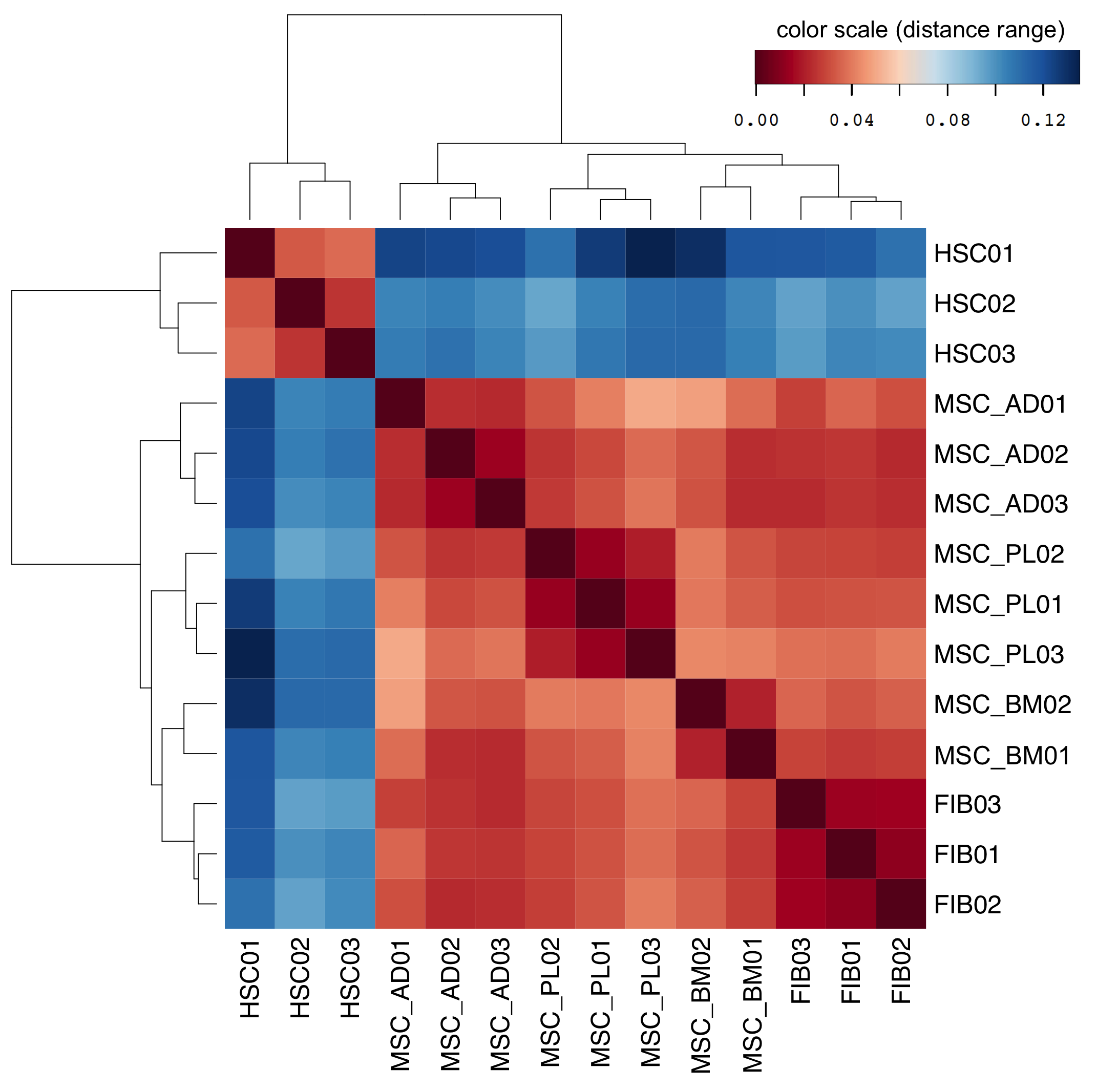

Supplement: Supplementary file 8 — Additional file 8: Figure S4: Comparative transcriptomic profiling of human MSCs versus related cell-types using genome-wide expression exon microarrays. MSCs samples isolated from three different tissue origins (bone marrow BM, placenta PL and adipose tissue AD) are compared with hematopoietic stem cells (HSC) and differentiated fibroblasts (FIB). The samples were analyzed using Affymetrix Human Exon 1.0 exon arrays, which have coverage for 20,238 unique human gene loci. The full expression signal of the arrays was normalized and calculated with RMA algorithm (using affy package from Bioconductor). Unsupervised hierarchical clustering of the global gene expression signatures was done to compare the samples. The heatmap shows the result of such clustering analysis. All genes were used for the distance calculations. The dendrogram of the sample clustering is also shown. Color scale provides a view of the distance range. (PNG 168 KB) [file 12864_2014_6787_MOESM8_ESM.png]

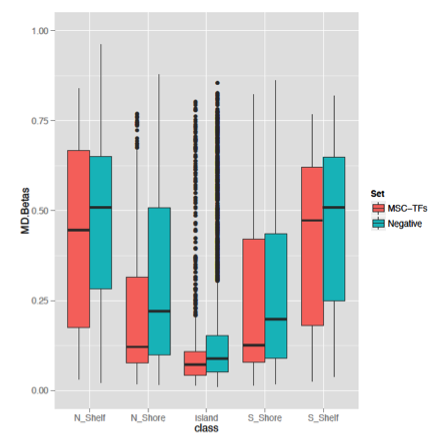

Supplement: Supplementary file 11 — Additional file 11: Figure S3: Analysis of the methylation data that is described in the manuscript. Boxplot of methylation distributions of the 135 pictured MSC-TFs (in red) compared to a negative set of other 135 TFs (in blue) not present in the MSC footprint. Different regions of the CpG islands were analyzed and shown in this plot. Beta values represent the methylation levels. (PNG 36 KB) [file 12864_2014_6787_MOESM11_ESM.png]
